# Wilms Tumor With Hepatic and Pulmonary Metastases in a Toddler: A Case Report

**DOI:** 10.7759/cureus.91176

**Published:** 2025-08-28

**Authors:** Khadija Elaitari, Nadia Boujida, Soukaina Jabour, Nazik Allali, Latifa Chat, Monim Ochan, Jawad Bouljrouf, Mounir Kisra, Maria El kababri, Amina Kili, Siham El haddad

**Affiliations:** 1 Department of Radiology, Children's Hospital, Rabat, MAR; 2 Department of Pediatric Surgery A, Children's Hospital, Rabat, MAR; 3 Department of Pediatric Hematology and Oncology, Children's Hospital, Rabat, MAR

**Keywords:** computed tomography (ct), diagnosis, management, metastasis, wilms tumor

## Abstract

Wilms tumor, also known as nephroblastoma, is the most common malignant renal tumor in children, typically diagnosed before the age of five. Diagnosis relies on a comprehensive clinical and radiological assessment to evaluate both local and distant tumor extension. We report the case of a 20-month-old female with Wilms tumor presenting initially with multiple pulmonary lesions and a solitary hepatic metastasis. Imaging, particularly computed tomography, plays a pivotal role in the accurate detection of these metastases, allowing precise staging and the planning of an appropriate therapeutic strategy. This case also highlights the importance of a multidisciplinary approach that integrates clinical, radiological, histopathological, and therapeutic data to optimize patient management.

## Introduction

Nephroblastoma, also known as Wilms tumor, is the most common malignant renal tumor in pediatrics, accounting for approximately 5% of all childhood cancers. This condition affects both boys and girls equally, with a mean age of onset of three years in sporadic cases and two years in hereditary forms [1]. It most often presents as a painless abdominal mass, sometimes accompanied by hematuria or hypertension [2].

Medical imaging plays a fundamental role throughout the management of Wilms tumor, enabling diagnosis, assessment of locoregional and distant spread, guidance of initial treatment, monitoring of therapeutic response, and detection of recurrence [3]. The therapeutic strategy is based on a multimodal approach, generally combining surgery, chemotherapy, and sometimes radiotherapy [2]. However, this case underscores specific diagnostic and therapeutic challenges, particularly in the preoperative setting, where accurate evaluation of vascular involvement is crucial for optimal surgical planning.

Approximately 15% of children with nephroblastoma present with distant metastases, either at initial diagnosis or at relapse. Metastases primarily affect the lungs and, less commonly, the liver [4,5].

## Case presentation

A 20-month-old girl presented with no relevant personal or family medical history. The medical history began four months earlier, when the patient’s mother incidentally palpated an abdominal mass in the left hypochondrium, which initially did not prompt medical consultation. The progression was characterized by rapidly increasing abdominal distension occurring one week before consultation, associated with fever, abdominal pain, and unquantified weight loss. There was no hematuria, bowel disturbances, or hypertension.

An abdominopelvic ultrasound was performed, revealing a large, well-defined, lobulated mass located on the left side of the abdomen and pelvis, with a heterogeneous echotexture. The mass had an iso-echoic tissue component containing hyper-echoic hemorrhagic areas and some cystic loculations. This mass extended beyond the midline and encompassed the abdominal vessels. The left kidney was not visualized, and a moderate amount of intraperitoneal fluid was present.

**Figure 1:**
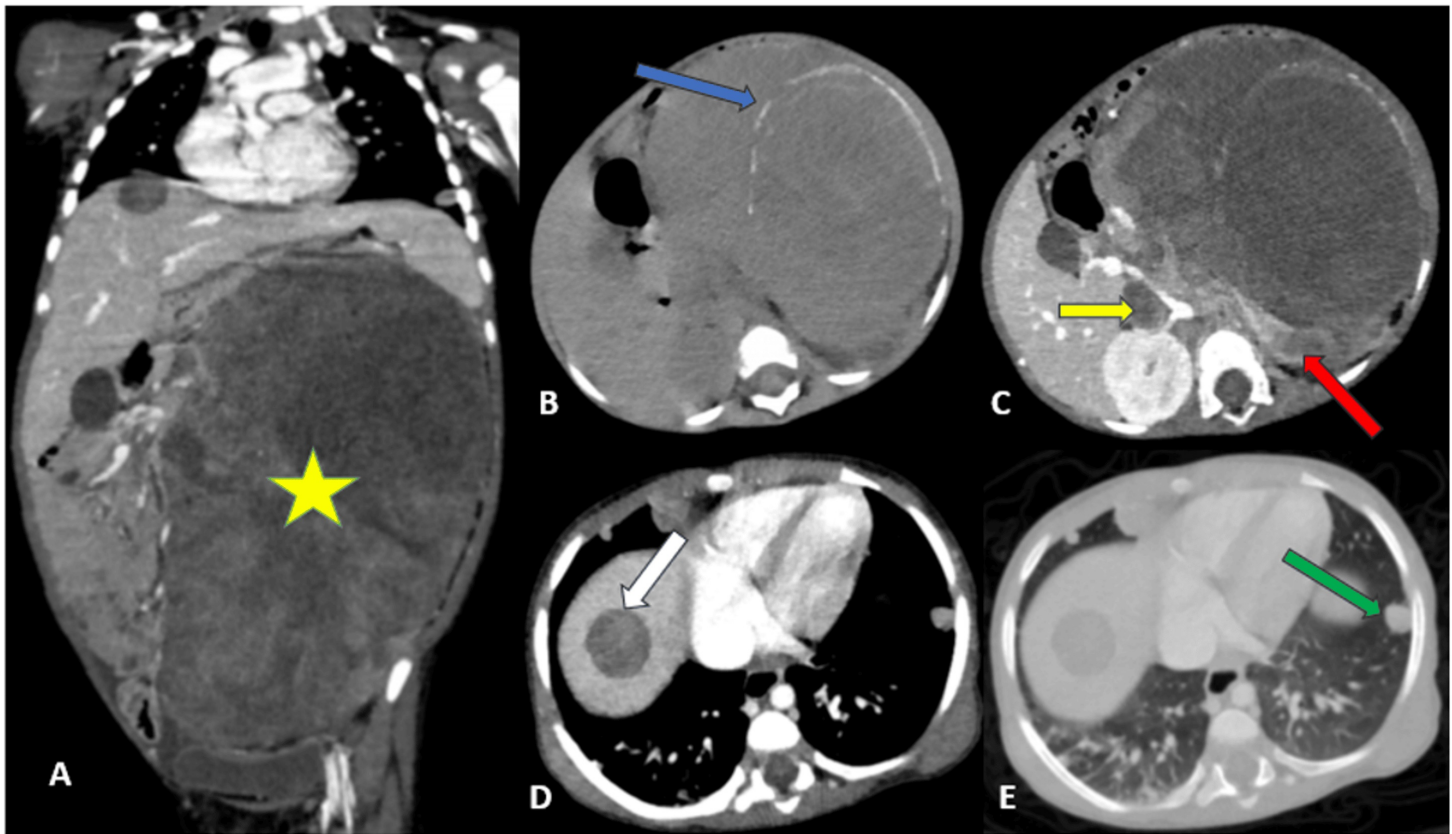
Thoraco-abdominopelvic CT scan with (A, C, D, E) and without (B) contrast enhancement, shown in coronal reconstruction (A) and axial sections (B, C, D, E). The images demonstrate a large left-sided retroperitoneal mass (asterisk) exhibiting the spur sign with the left kidney (red arrow), containing internal calcifications (blue arrow), and showing heterogeneous, low enhancement. The scan also reveals an intravascular tumor thrombus within the IVC (yellow arrow), as well as pulmonary (green arrow) and hepatic metastases (white arrow). IVC, inferior vena cava

A thoraco-abdominopelvic CT scan was requested for better characterization of the mass and to assess locoregional and distant extension. It revealed a large retroperitoneal tumor on the left side, extending into the abdomen and pelvis, well-circumscribed with lobulated contours and heterogeneous density. It consisted of three components: arc-shaped calcifications, a liquid portion, and a tissue portion that showed heterogeneous enhancement after contrast administration. The tumor produced the *spur sign* at the superior pole of the left kidney. This feature supports the renal origin of the mass and helps in differentiating Wilms tumor from other retroperitoneal neoplasms such as neuroblastoma. The presence of calcifications is more frequently associated with neuroblastoma, whereas they are uncommon in Wilms tumor, thus representing an atypical but important feature in this case. The mass measured 18 cm in height, encompassed the homolateral renal pedicle with no clear visualization of the left renal vein, extended beyond the midline, compressed the inferior vena cava (IVC), which contained a thrombus measuring 20 mm in length, and infiltrated the left diaphragmatic crus. A hepatic mass located in segment VIII, with the same density as the renal tumor, corresponded to a secondary lesion. Thoracic imaging showed multiple bilateral pulmonary nodules with a *cannonball *appearance, suggestive of metastases (Figure 1).

Given the presence of a renal mass with secondary pulmonary and hepatic lesions, histological confirmation was considered necessary. The patient underwent an ultrasound-guided biopsy of the renal mass. Histopathological examination revealed a triphasic tumor proliferation consisting of a mesenchymal component with primitive mesenchyme and rhabdomyoblastic proliferation, an epithelial component of immature tubules, and a blastemal component composed of nests of undifferentiated cells. This morphological pattern is compatible with a triphasic nephroblastoma.

**Figure 2:**
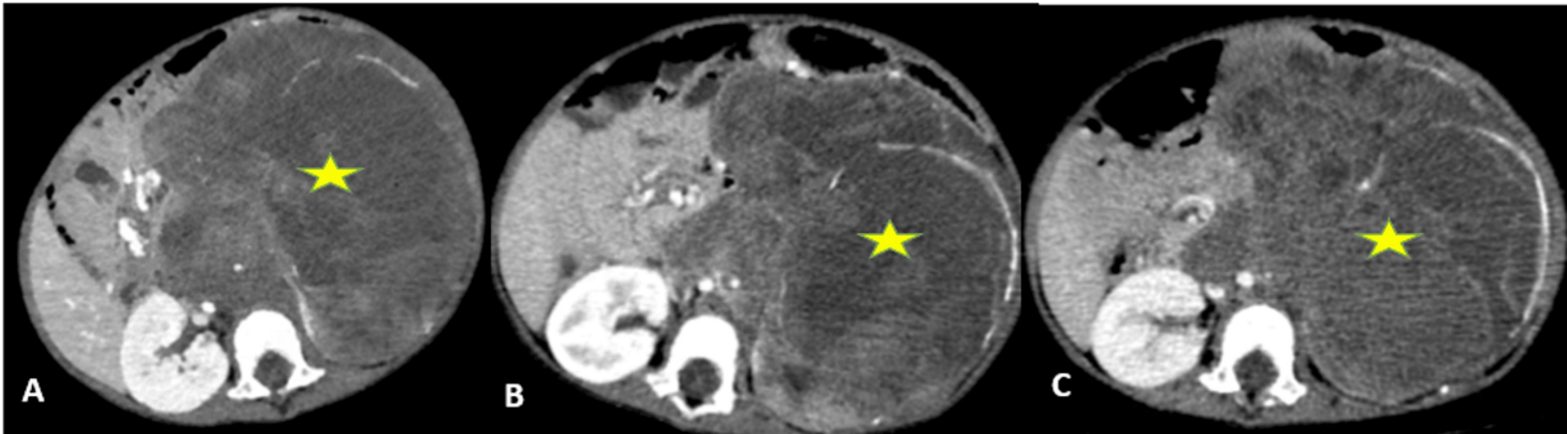
Axial slices of the initial abdominopelvic CT scan (A), first follow-up (B), and second follow-up (C) images of the retroperitoneal mass (asterisk), showing a slight size reduction.

**Figure 3:**
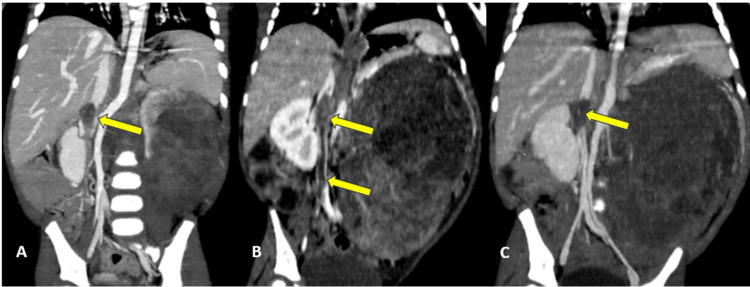
Coronal reconstructions of the initial abdominopelvic CT scan (A), first follow-up (B), and second follow-up (C) images showing the evolution of the intravascular thrombus within the IVC (yellow arrow). IVC, inferior vena cava

The child received neoadjuvant chemotherapy for 12 weeks, with two CT follow-up scans performed at the 5th and 12th weeks of treatment. The first follow-up scan showed a slight regression of the renal mass by 7% (Figures 2A-2B), with increased extension of the caval thrombus reaching the iliac veins (Figures 3A-3B). The second follow-up scan demonstrated a 12% regression of both the tumor (Figures 2B-2C) and the thrombus (Figures 3B-3C) compared with the first follow-up scan. Pulmonary and hepatic metastases remained stable on both follow-up exams.

**Figure 4:**
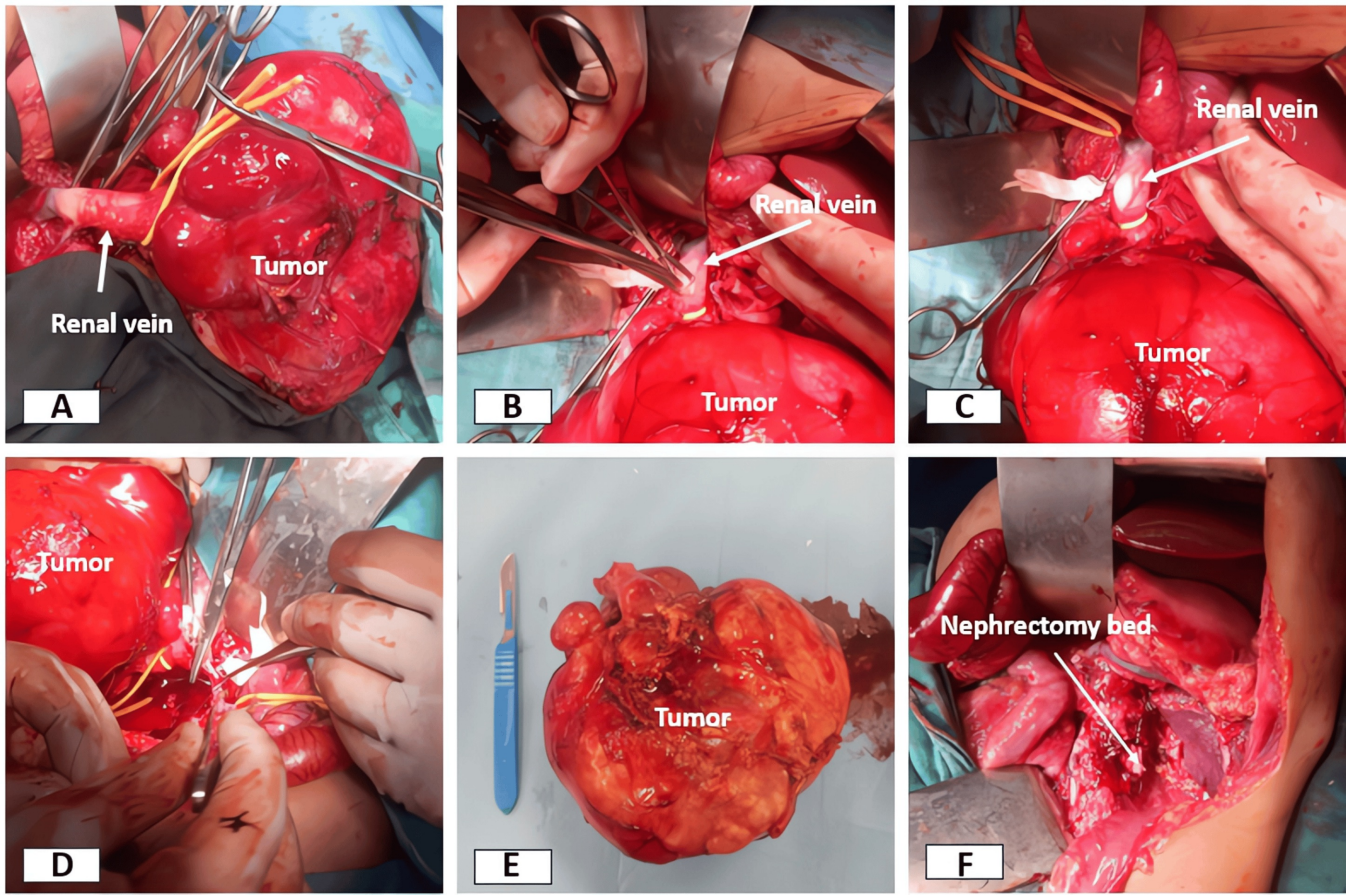
Intraoperative images showing the main steps of the extended left nephroureterectomy with adrenalectomy, including isolation of the tumor and left renal pedicle (A), longitudinal venotomy of the renal vein (B-C), and en bloc extraction of the intravascular tumor thrombus with the tumor mass (D-E).

The patient underwent an extended left nephroureterectomy with adrenalectomy (Figure 4) via a transverse supraumbilical laparotomy. Intraoperative exploration revealed a large encapsulated retroperitoneal mass originating from the left kidney, crossing the midline, and intimately related to the diaphragm, pancreas, stomach, and left liver. The ipsilateral adrenal gland was included within the tumor mass. A strong adhesion to the left border of the aorta was also noted.

Progressive dissection of the mass allowed isolation of the left renal pedicle. The markedly dilated left renal vein was completely obstructed by a tumor thrombus extending to its junction with the IVC (Figure 4A). The IVC demonstrated aplasia of its subrenal segment, limiting venous return. It was clamped distal to the left renal vein with a Satinsky clamp, allowing a longitudinal venotomy of the renal vein (Figures 4B-4C). The thrombus was then extracted en bloc, in continuity with the tumor (Figures 4D-4E).

The procedure continued with ligation and division of the ureter at the juxtavesical level, allowing complete excision without tumor rupture. A hilar, latero-aortic, and mesenteric lymph node dissection was performed for staging purposes. Hemostasis was carefully verified, and a Redon drain was placed in the renal lodge to drain the operative cavity.

Postoperatively, the patient tolerated the procedure well. The management plan includes continuation of adjuvant chemotherapy according to the current protocol, with scheduled postoperative imaging to monitor the evolution of pulmonary and hepatic metastatic lesions as well as the tumor bed. The patient is currently under multidisciplinary pediatric oncology follow-up.

## Discussion

General information

Nephroblastoma, or Wilms tumor, accounts for more than 90% of malignant renal tumors in children. It generally has a good prognosis, with a survival rate exceeding 90% for localized forms and close to 70% for those with metastases [2]. In most cases, it is unilateral and solitary; however, about 5% to 7% of children develop bilateral involvement, and 10% present multiple tumor foci in the same kidney. Children with a genetic predisposition (Beckwith-Wiedemann, Denys-Drash, or WAGR syndromes) have an increased risk of multifocal or bilateral forms [3]. Among children with metastatic Wilms tumor, hepatic metastases occur in approximately 20% of cases. Reported survival rates vary widely, ranging from 13% to 89%, with generally poorer outcomes for patients presenting with metachronous hepatic metastases compared to synchronous lesions [5].

Clinical presentation

In children, Wilms tumor most often presents as a painless abdominal mass, causing abdominal distension, found in about 75% to 95% of cases [1,2,6]. Associated symptoms such as abdominal pain, fatigue, or hematuria (microscopic or macroscopic) are present in 20% to 30% of cases, while arterial hypertension is observed in about 25% of patients [1,6]. In some cases, the presentation is more acute, with a rapid increase in abdominal volume, accompanied by anemia, fever, severe pain, or severe hypertension, often related to subcapsular hemorrhage of the tumor [6].

Radiological manifestations

In children, abdominal ultrasound is the first-line examination in the presence of an abdominal mass, allowing confirmation of a retroperitoneal mass and investigation of its origin, although this may be difficult in cases of large masses [3,6]. In our case, ultrasound did not confirm the renal origin due to the significant size of the mass. It also allows evaluation of the liver and vessels to look for the possible presence of an intravascular tumor thrombus [6].

After identification of the mass, a locoregional and distant extension assessment with CT or MRI is essential for better characterization. The choice between these modalities depends on local practice, as both offer comparable effectiveness for abdominopelvic staging. The mass shows the spur sign with the kidney, lower enhancement than the healthy parenchyma, and heterogeneity due to areas of necrosis or hemorrhage [3].

CT is more effective than ultrasound in detecting an intravascular tumor thrombus, present in about 11% of Wilms tumor cases, with extension to the IVC in 6% of cases. The presence of a thrombus in the renal vein should be checked intraoperatively by palpating the vein before ligation and section. Chest CT is the examination of choice for detecting pulmonary metastases, while other rare metastatic sites, such as the liver, are usually well assessed by the initial abdominopelvic CT scan [6].

In this case, CT proved to be the most effective imaging modality for analyzing the mass, revealing internal calcifications and providing a detailed assessment of local and distant extension, including detection of the intravascular tumor thrombus as well as pulmonary and hepatic metastases. These findings, combined with an atypical radiologic presentation according to International Society of Pediatric Oncology (SIOP) criteria, prompted the performance of a biopsy of the mass and guided therapeutic management.

Finally, MRI is recommended in cases of bilateral tumors or known predisposition, particularly with diffusion sequences and high-resolution post-contrast images, which facilitate the detection of small synchronous tumors or nephrogenic rests [3].

Histology and prognosis

Wilms tumor is classically defined as a triphasic tumor, comprising stromal, epithelial, and blastemal elements, although the simultaneous coexistence of all three components is not always necessary to confirm the diagnosis. According to the National Wilms Tumor Study (NWTS) classification, Wilms tumors are divided into distinct histopathological types based on their prognosis: those with a favorable prognosis, lacking anaplasia, and those with an unfavorable prognosis, characterized by anaplasia, defined by marked nuclear and cytological atypia, which may be focal or diffuse [6].

Wilms tumor is classified into five stages according to a surgical-pathological staging system, which is essential for determining the therapeutic strategy and assessing the prognosis [2]. In our case, the patient was classified as stage IV due to the presence of distant metastases.

The prognosis of nephroblastoma depends on several clinical factors, including tumor stage, histological type, presence of metastases at diagnosis, and patient age. An advanced stage or the presence of metastases is associated with an unfavorable prognosis. Furthermore, children older than 10 years generally have a less favorable prognosis [2].

Management

Treatment is primarily based on surgery, usually a radical nephrectomy with lymph node dissection. In cases of bilateral tumors, a partial nephrectomy can be performed to preserve renal function. Surgery is followed by adjuvant chemotherapy, with intensive protocols for aggressive or advanced forms. Radiotherapy is reserved for advanced stages or in cases of distant metastases, especially pulmonary [2].

The National Wilms Tumor Study Group (NWTSG) recommends an initial radical nephrectomy for accurate staging and histological assessment, followed by adjuvant chemotherapy. In contrast, SIOP advocates for neoadjuvant chemotherapy before surgery to reduce tumor size and the risk of intraoperative complications, particularly tumor spillage, especially in patients older than six months. This approach is particularly useful in cases of large tumors or vascular involvement, facilitating more complete resection [2,6].

Surgical excision of the intravascular thrombus is recommended when technically feasible. In metastatic disease, treatment of pulmonary metastases relies on chemotherapy and, if necessary, radiotherapy; pulmonary resection is rarely indicated, as chemotherapy is generally effective [6].

Some studies indicate that resection of hepatic metastases could improve survival. However, the lack of reliable data on the effectiveness of this intervention makes it difficult to formulate precise recommendations [5].

After treatment for Wilms tumor, structured follow-up is recommended to detect local and distant recurrence, including chest CT for pulmonary surveillance and abdominal ultrasound, with particular attention to the contralateral kidney [1].

## Conclusions

Wilms tumor is a pediatric malignancy with a generally favorable prognosis, but metastatic forms, particularly those with hepatic involvement, remain associated with a poor prognosis. Imaging plays a crucial role in assessing locoregional extension (including vascular thrombi) and distant metastases, allowing for precise staging and tailored therapeutic planning, highlighting the importance of a multidisciplinary approach.
